# Hypoxemia-induced TIGIT expression in obstructive sleep apnea is reversible with continuous positive airway pressure

**DOI:** 10.3389/fimmu.2026.1769874

**Published:** 2026-03-10

**Authors:** Paula Pérez-Moreno, Elena Díaz-García, Cristina López-Fernández, Aldara García-Sánchez, Eva Mañas, Laura Pozuelo-Sánchez, María Torres-Vargas, Elisabet Martínez-Cerón, Raquel Casitas, Raúl Galera, María Fernández Velasco, Luis del Peso, Francisco García-Río, Carolina Cubillos-Zapata

**Affiliations:** 1 Biomedical Research Networking Centre on Respiratory Diseases (CIBERES), Madrid, Spain; 2 Respiratory Diseases Group, Respiratory Diseases Department, Hospital La Paz Institute for Health Research – IdiPAZ, Madrid, Spain; 3 Faculty of Medicine, Autonomous University of Madrid, Madrid, Spain; 4 Servicio de Neumología, Hospital Universitario Ramón y Cajal, Madrid, Spain; 5 Clinical and Invasive Cardiology Research Group (ICCI-PAZ), Hospital La Paz Institute for Health Research– IdiPAZ, Madrid, Spain; 6 Cardiovascular Biomedical Research Centre Network (CIBERCV), Madrid, Spain; 7 Instituto de Investigaciones Biomédicas Sols-Morreale (IIBM), Consejo Superior de Investigaciones Científicas, Universidad Autónoma de Madrid, Madrid, Spain

**Keywords:** HIF-1α, hypoxemia, immune checkpoints, immune surveillance, OSA, T-cells, TIGIT

## Abstract

**Introduction:**

Obstructive Sleep Apnea (OSA) is a prevalent syndrome characterized by intermittent hypoxemia and elevated risk of comorbidities, including cancer. In this context, the immune response may contribute to tumor evasion though immune checkpoints. Herein, we investigate the TIGIT immune checkpoint in OSA patients and its association with hypoxemia.

**Methods:**

We recruited 94 severe OSA patients without cancer evidence and 92 control subjects to study the TIGIT receptors and their ligands in T cells and monocytes, respectively. Furthermore, we examined the role of hypoxemia – particularly the involvement of HIF-1α (hypoxia inducible factor-1α) - using a combination of *in vitro* models. Moreover, we evaluated the effect of one year of standard therapy with CPAP (continuous positive airway pressure) in OSA patients.

**Results:**

Our data suggests that the TIGIT expression increase on T cells from OSA patients and is associated with clinical indicators of hypoxemia. *In vitro* hypoxemia models confirm the role of HIF-1α in the upregulation of TIGIT expression. However, within the OSA cohort without evidence of cancer, we did not detect significant differences in TIGIT ligands, either in their membrane-bound or soluble forms. Importantly, one year of CPAP treatment reduce the TIGIT expression.

**Conclusions:**

Hypoxemia in OSA patients increases TIGIT expression, contributing to a T-cell exhaustion phenotype. CPAP treatment reduces TIGIT expression on T lymphocytes. Altogether, these findings highlight the impact of hypoxemia effect on immune response, which may help explain the high cancer incidence in OSA patients.

## Introduction

Obstructive sleep apnea (OSA) is a syndrome characterized by the partial or complete obstruction of the upper airways during sleep, leading to intermittent hypoxia (IH), fragmented sleep, and increased inspiratory effort (1). These physiological disturbances are associated with a higher risk of various comorbidities, including cancer, cardiovascular, and metabolic disorders (2–4). In fact, the prevalence of cancer in patients with OSA is 1.53 times higher than in individuals without OSA (5, 6). The elevated risk of comorbidities associated with OSA highlights the need to investigate its underlying molecular mechanisms, which could aid in predicting disease severity and preventing secondary complications. In this context, immune checkpoints (IC) may be responsible for the disruption of immune surveillance, leading to OSA-associated diseases (7, 8).

Immune checkpoints modulate immune responses by regulating the activity of various immune cell populations, including T lymphocytes (9–11). IH further disrupts T-cell function by upregulating several immune checkpoint pathways, including the adenosine-producing enzymes CD39/CD73, the PD-1/PD-L1 axis, the inhibitory receptor PSGL-1 (P-selectin glycoprotein ligand-1) and the TIM-3/Galectin-9 axis (7, 12–15). Along these lines, we focus on TIGIT, an immune checkpoint that has been studied in recent years in the field of tumor immunotherapy (16) and plays important roles in immune modulation (17). TIGIT (T cell immunoglobulin and ITIM domain protein), also known as WUCAM, Vstm3, and VSIG9 (18, 19) is an inhibitory receptor belonging to the immunoglobulin superfamily. TIGIT is part of a regulatory complex in T cells that includes other inhibitory receptors, such as CD96 and CD112R, as well as costimulatory receptors like CD226 (17, 20, 21). This receptor complex modulates the immune response through competition for the same ligand, wherein the inhibitory receptor has higher affinity, whereas the activating receptor has lower affinity (22). The binding of TIGIT to its ligands CD155, CD112, and CD113 on antigen-presenting cells (APCs) or tumor cells triggers a signaling cascade that ultimately inhibits the production of certain cytokines, such as IL-2, TNF-α, and IFN-γ, leading to T cell dysfunction (17, 23). Among TIGIT ligands, CD155 (PVR, poliovirus receptor) and CD112 (nectin-2, PVRL2) exhibit the highest affinity (24, 25). Additionally, they are involved in contact inhibition, proliferation, and cell survival, functions with significant implications for tumor progression (26, 27).

Given the established association between obstructive sleep apnea (OSA) and immune dysregulation, in this study we aim to investigate the expression of TIGIT in T cells from patients with OSA and to elucidate the potential mechanisms driving its regulation. Specifically, we hypothesize that intermittent hypoxia characteristic of OSA promotes TIGIT overexpression in T lymphocytes via HIF-1α-mediated pathways, contributing to T cell dysfunction. Furthermore, we propose that this upregulation may be reversed by effective OSA treatment, such as continuous positive airway pressure (CPAP) therapy.

## Methods

### Study design and participants

This investigation included 94 consecutive patients diagnosed with severe obstructive sleep apnea (OSA group); the first 36 patients from this group who completed one year of CPAP treatment (post-CPAP group); and 92 control subjects (non-OSA group). For additional details, please refer to the **Supplementary Methods**. In brief, OSA was diagnosed using respiratory polygraphy (Embletta GOLD, ResMed), which monitored respiratory effort, oronasal airflow, heart rate, chest and abdominal movement, and oxygen saturation (SaO_2_) continuously. Patients were categorized as having severe OSA if their apnea-hypopnea index (AHI) exceeded 30 events/h. Control subjects, matched by age and sex with OSA patients, were selected from the census of the Madrid metropolitan area in Spain. Respiratory polygraphy confirmed the absence of OSA in the non-OSA group. The study was approved by the local ethic committee (PI-3646) and informed consent was obtained from all participants.

### Peripheral blood mononuclear cell isolation

Peripheral blood mononuclear cells (PBMCs) were isolated by centrifugation using a Ficoll-Paque Plus density gradient (*Amersham Bioscience, Uppsala, Sweden*). Subsequently, 5 × 10^6^ PBMCs were seeded into each well of 6-well plates. The cells were cultured in Roswell Park Memorial Institute (RPMI) 1640 medium supplemented with 100 U/mL penicillin and 100mg/mL streptomycin, along with 10% fetal bovine serum (FBS). Finally, the cells were incubated for 16 hours at 37 °C in a 5% CO_2_ atmosphere.

### Flow cytometry

After a 16-hour incubation, cells were collected. For intracellular staining (perforin, granzyme B, T-bet, and TOX), cells were fixed for 10 minutes and washed twice with Perm/Wash buffer for permeabilization using the BD Cytofix/Cytoperm™ kit (BD Biosciences, Eysins, Switzerland). All samples were stained for 30 minutes at 4 °C in the dark using the anti-human antibodies listed in **Supplementary Table 1**. The cells were subsequently washed with phosphate-buffered saline (PBS) supplemented with 1% fetal bovine serum (FBS). Finally, the samples were analyzed using a BD FACS-Celesta flow cytometer (BD Biosciences, Eysins, Switzerland), and the data were analyzed using FlowJo vX.0.7 software (FlowJo, USA) (**Supplementary Figure 1**).

### Determination of plasma levels of TNF-α and IFN-γ

Plasma samples were analyzed for TNF-α and IFN-γ concentrations using a customized bead-based multiplex assay (LEGENDplex™ Human Inflammation Panel 1; BioLegend, Inc., San Diego, CA, USA), according to the manufacturer’s instructions. The detection limits were 3.6 pg/mL for soluble TNF-α and 2.9 pg/mL for soluble IFN-γ.

### Intermittent hypoxia model

Intermittent hypoxia *in vitro* experiments were performed using an incubation chamber connected to a computer-controlled oxygen/nitrogen controller, utilizing the BioSpherix OxyCycler C42 system (Redfield, NY, USA). This system facilitates cyclical fluctuations in oxygen levels while maintaining stable CO_2_ concentrations, precisely regulating the gas composition within each chamber as we have previously described (15, 28, 29).

### HIF-1α inhibition and stimulation assay

To inhibit or stimulate Hypoxia-Inducible Factor 1-alpha (HIF-1α), two distinct approaches were utilized. First, PBMCs were treated with 30 µM PX-478 (MedKoo Biosciences, Morrisville, NC, USA) for 16 hours, as previously described (15, 30). Additionally, for the dimethyloxallyl glycine (DMOG) assay, cells were treated with 100 µM DMOG for 16 hours under standard culture conditions (12).

### Statistical analysis

Demographic and clinical characteristics were reported as mean ± standard deviation for continuous variables, and as frequencies accompanied by their respective percentages for categorical variables. Group comparisons were executed using the Mann-Whitney U test, two-way ANOVA with *post hoc* Tukey’s or Bonferroni tests, or Wilcoxon test, depending on the nature and distribution of the variables. Correlations between continuous variables were assessed using Spearman’s rank correlation. The Anderson-Darling and D’Agostino-Pearson tests were utilized to evaluate data distribution. A significance level of p < 0.05 was applied for all analyses. Statistical analyses were performed using Prism 8.0 software (GraphPad, USA).

## Results

### Patient characteristics

In total, 94 patients with severe OSA were included who had been recently diagnosed (OSA group), 92 control subjects (non-OSA group). As shown in **Table 1**, there were no significant differences between the non-OSA and OSA groups in terms of sex, age, or Epworth Sleepiness Scale score.

**Table 1:** Main characteristics of study subjects.

Demographic, Clinical, and Polysomnographic Characteristics	Non-OSA (n=92)	OSA group (n=94)	P-valor
Sex, n (%) male	56 (60,86%)	59 (62.76%)	>0.999
Age (y)	53,35 (11,40)	55,26(11,21)	0,075
BMI (kg/m2)	26,50 (4,23)	33,41 (6,61)	<0,0001
Smoking, n (%)	8 (8,69%)	21 (22,34%)	0,025
AHI (events/h)	4,04 (5,49)	51,57 (18,35)	<0,0001
ODI (events/h)	6,21 (8,38)	51,87 (19,36)	<0,0001
Mean SaO2 (%)	93,47 (1,81)	89,98 (2,61)	<0,0001
Min SaO2 (%)	83,03 (14,89)	73,43 (8,57)	<0,0001
Epworth sleepiness score	7,86 (4,15)	8,61 (4,89)	0,537

BMI, body mass index; AHI, apnea-hypopnea index; ODI, oxygen desaturation index; SaO2, oxyhemoglobin saturation.

### TIGIT expression is increased in patients with OSA on T cell membranes

**Figure 1:**
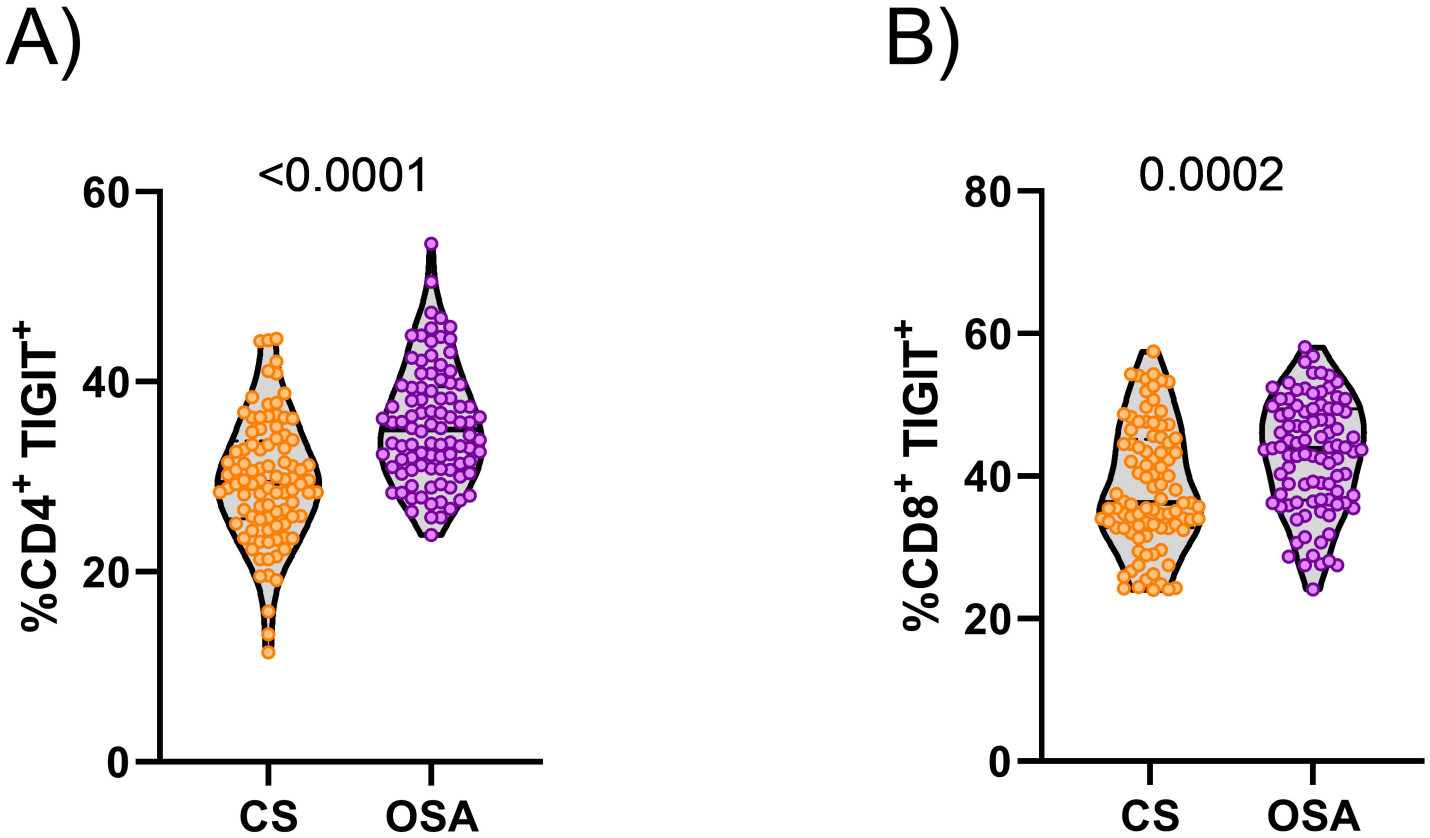
TIGIT expression on T-lymphocyte. **(A)** Violin plots showing the percentage of T-lymphocytes CD4^+^ that express TIGIT determined by flow cytometer in randomly selected control subjects (n=92) and OSA patients (n=94). **(B)** Violin plots showing the percentage of T-lymphocytes CD8^+^ that express TIGIT determined by flow cytometer in randomly selected control subjects (n=92) and OSA patients (n=94). Comparisons were assessed by used Mann-Whitney test, p-values are shown.

**Figure 2:**
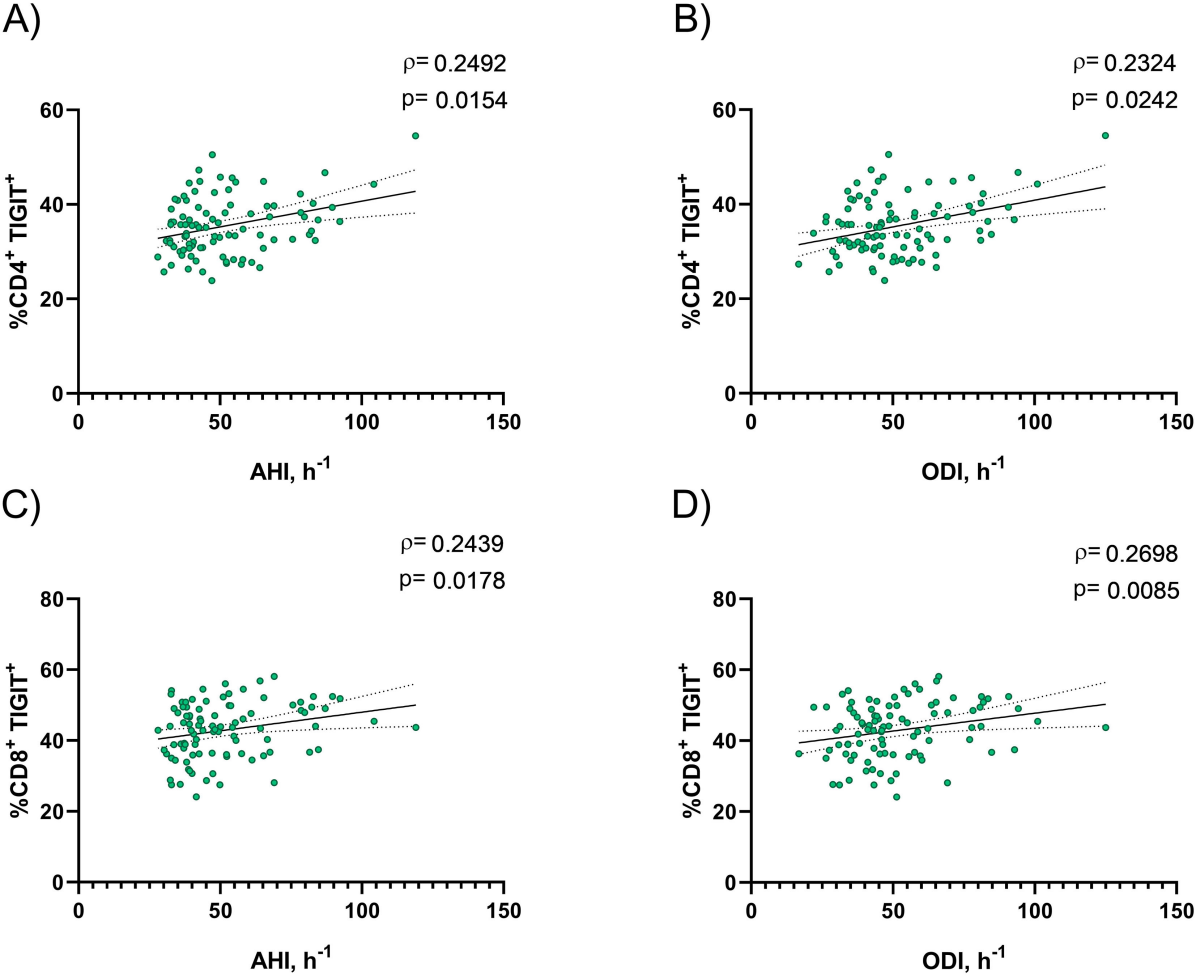
Correlation between TIGIT expression and clinical parameters related with OSA severity. Association between the percentage of TIGIT expression on CD4^+^**(A, B)** and CD8^+^**(C, D)** T lymphocytes and **(A–C)** apnea-hypopnea index [AHI] (n=94) and **(B–D)** oxygen desaturation index [ODI] (n=94). Spearman’s correlation coefficients (ρ) and p-values (p) are shown. The solid line represents the regression line, and dotted lines represents the 95% CI.

The analysis of TIGIT expression on the membrane of CD4^+^ and CD8^+^ T lymphocytes revealed a significant increase in patients with OSA compared to control subjects (**Figure 1**). However, the expression levels of its ligands, CD155 and CD112 (**Supplementary Figure 2**), as well as its soluble form (**Supplementary Figure 3**), did not show significant differences between the two groups. Additionally, we investigated the association between TIGIT expression and hypoxemia clinical parameters related to OSA to better understand the impact on immune checkpoint regulation. The clinical parameters apnea–hypopnea index (AHI) and oxygen desaturation index (ODI) were significantly correlated with TIGIT expression in both CD4^+^ and CD8^+^ T cells (**Figure 2**). These findings indicate a relationship between hypoxemia OSA severity and TIGIT expression on T lymphocytes.

### TIGIT expression on T cells increased by intermittent hypoxia

**Figure 3:**
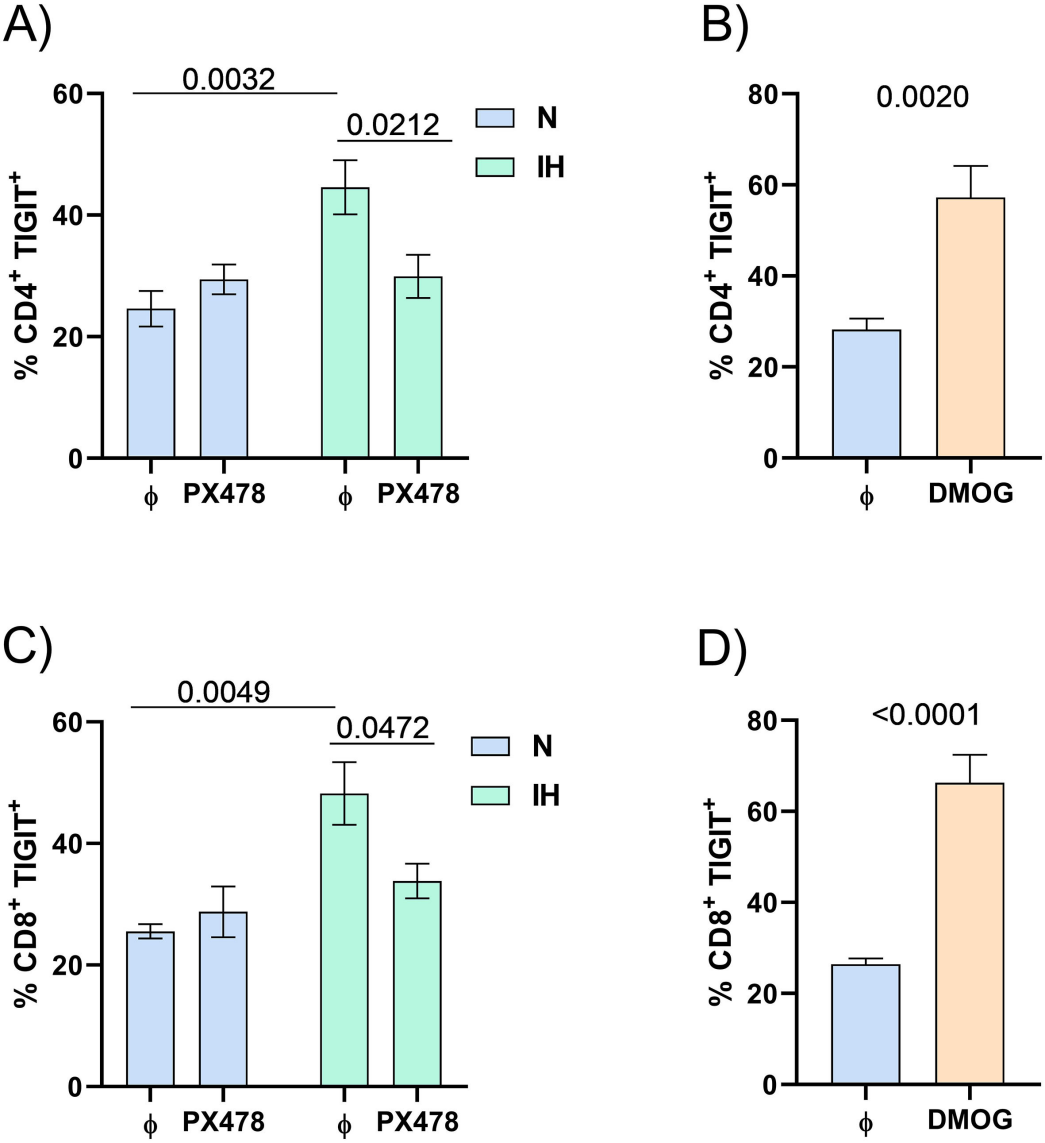
TIGIT expression on T lymphocytes from PBMCs of healthy donors (n = 10). **(A, C)** PBMCs were cultured under normoxia **(N)** or intermittent hypoxia (IH) conditions and treated or not with a specific HIF-1α inhibitor (PX-478, 30 μM) for 16 hours. The percentage of CD4^+^**(A)** or CD8^+^**(C)** T lymphocytes expressing TIGIT was assessed by flow cytometry. Group comparisons were performed using two-way ANOVA followed by Tukey’s *post hoc* test for multiple comparisons. **(B, D)** PBMCs from healthy donors (n = 10) were cultured under normoxia conditions in the presence or absence of DMOG (100 μM), an inhibitor of HIF-1α degradation under normoxia, for 16 hours. The percentage of CD4^+^**(B)** or CD8^+^**(D)** T lymphocytes expressing TIGIT was measured by flow cytometry. Comparisons were conducted using the Mann–Whitney test; p-values are reported.

Given that intermittent hypoxia is a hallmark of OSA, we explored whether TIGIT expression is increased in T lymphocytes exposed to intermittent hypoxia (IH) conditions. To address this, we established an intermittent hypoxia *in vitro* model using PBMCs from healthy donors for 16 hours. Our results showed an upregulation of TIGIT expression on both CD4^+^ and CD8^+^ T cells under IH model (**Figures 3A, C**). Based on these results, we examined whether the increase in TIGIT expression could be attributed to HIF-1α–mediated hypoxemia. To investigate this, we conducted two complementary *in vitro* experiments: one involving inhibition and the other overexpression of HIF-1α. HIF-1α inhibition was achieved using PX-478 under intermittent hypoxia conditions, while overexpression was induced under normoxic conditions using DMOG, a compound that stabilizes HIF-1α by inhibiting its degradation. The data demonstrated that TIGIT expression was suppressed in both CD4^+^ and CD8^+^ T cells when HIF-1α was inhibited during intermittent hypoxia (**Figures 3A, C**). Conversely, overexpression of TIGIT was observed when cells were treated with DMOG (**Figures 3B, D**). These results corroborate the HIF-1α role on the TIGIT expression on CD4^+^ and CD8^+^ T lymphocytes.

### TIGIT upregulation is associated with T cell exhaustion

**Figure 4:**
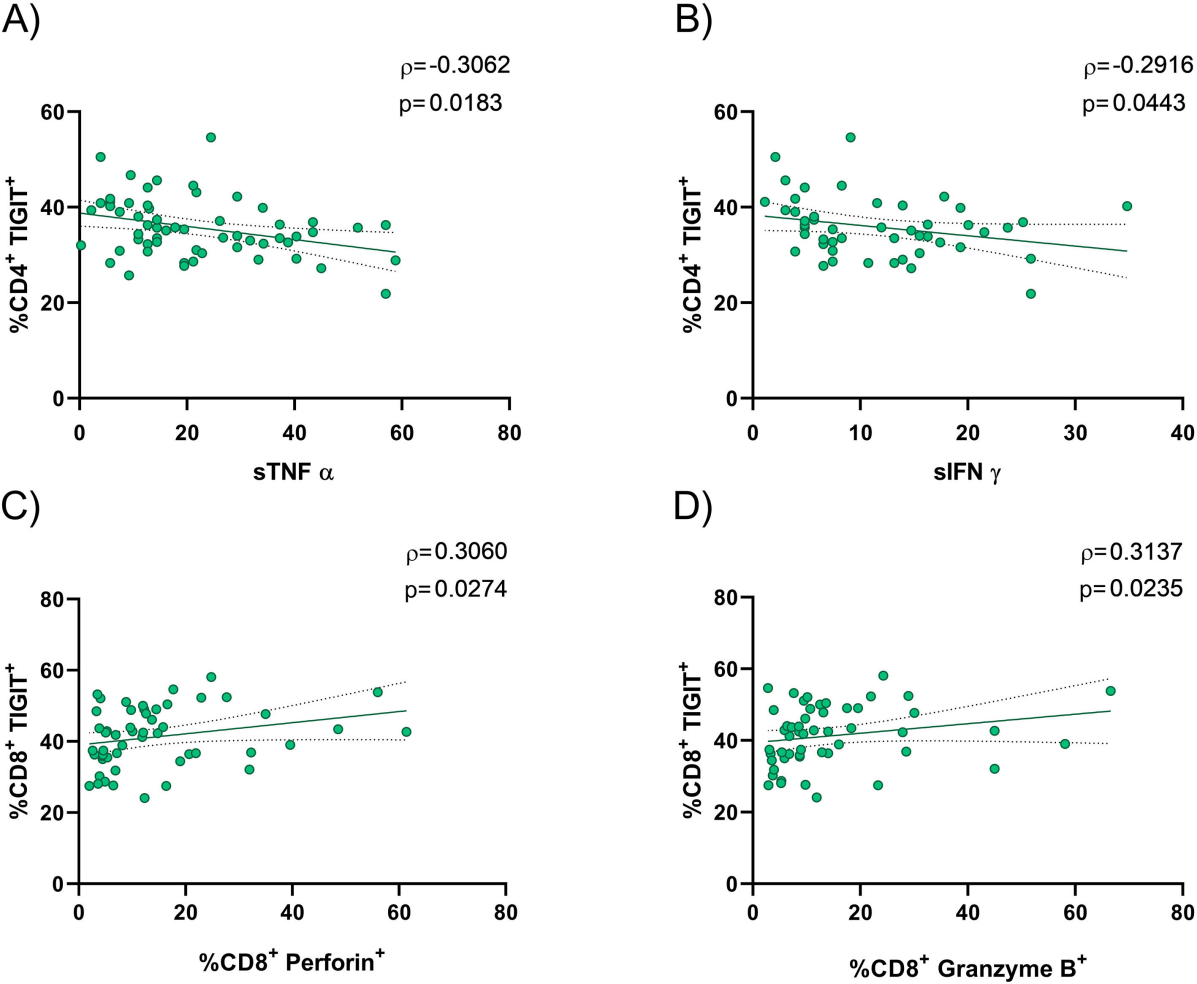
Association between TIGIT expression on T lymphocytes and functional markers. **(A, B)** Spearman rank correlation between TIGIT expression on CD4^+^ T lymphocytes and soluble TNF-α (n=59) **(A)** and soluble IFN-γ (n=48) **(B)**. **(C, D)** Spearman rank correlation between TIGIT expression on CD8^+^ T lymphocytes and perforin (n=52) **(C)** and granzyme B expression (n=52) **(D)**. The solid line represents the regression line, and the dotted lines indicate the 95% confidence interval.

**Figure 5:**
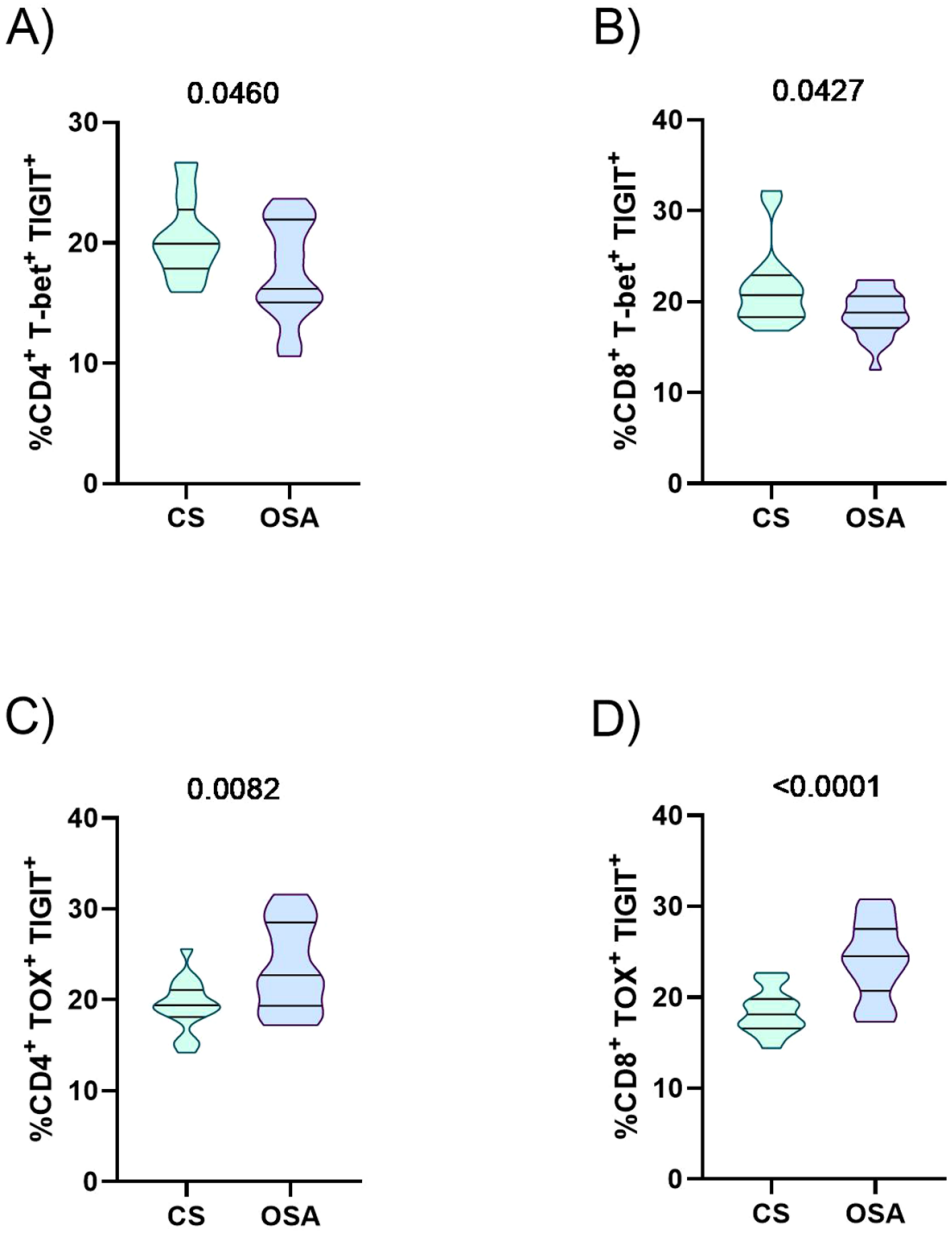
Transcription factors and TIGIT expression on T lymphocytes. **(A, B)** Violin plots illustrating the percentage of CD4^+^**(A)** and CD8^+^**(B)** T cells expressing T-bet and TIGIT, as assessed by flow cytometry, in control subjects (n=16) and OSA patients (n=21). **(C, D)** Violin plots illustrating the percentage of CD4^+^**(C)** and CD8^+^**(D)** T cells expressing TOX and TIGIT, as assessed by flow cytometry, in control subjects (n=16) and OSA patients (n=21). Statistical differences between groups were analyzed using the Mann–Whitney test, with p-values indicated.

The physiological disturbances caused by OSA are linked to an increased risk of cancer. These alterations may be driven by dysregulation of T cell functional markers. Therefore, we assessed whether elevated TIGIT expression in OSA patients is associated with changes in the expression of key T cell functionality markers. As shown in (**Figures 4A, B**), there was a negative correlation between TIGIT expression on CD4^+^ T cells and plasma levels of immune mediators TNF-α and IFN-γ. In contrast, no correlation was observed between CD8^+^ T cell TIGIT expression and these cytokines (**Supplementary Figure 4**). Moreover, the percentage of CD8^+^ T cells expressing TIGIT was associated with the expression of perforin and granzyme (**Figures 4C, D**). In addition, we examined transcription factors associated with the T-cell exhaustion phenotype to assess the functional capacity of T cells. Analysis of **Figures 5A, B** indicates that T-bet expression in TIGIT^+^ CD4^+^ and CD8^+^ T lymphocytes are decreased in OSA patients compared with control subjects (CS). Conversely, expression of the transcription factor TOX in TIGIT^+^ CD4^+^ and CD8^+^ T cells is increased in OSA patients compared with CS (**Figures 5C, D**). These findings suggest a dysregulation in T cell effector function in OSA patients with elevated TIGIT expression.

### TIGIT expression decrease after CPAP treatment

**Figure 6:**
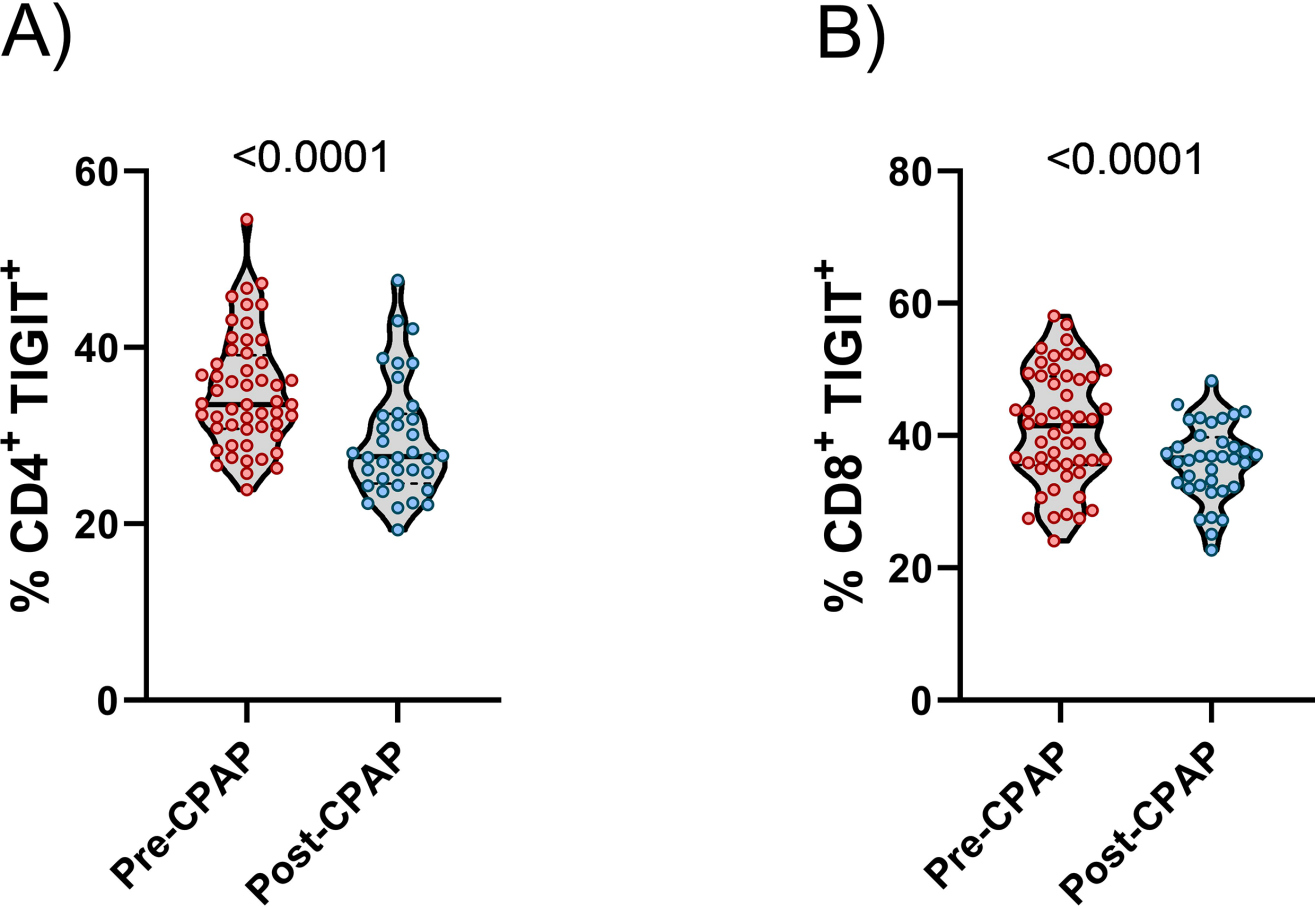
TIGIT expression on T-cells pre and post CPAP treatment. **(A)** Violin plots illustrating the percentage of CD4^+^ T lymphocytes expressing TIGIT, as assessed by flow cytometry, in OSA patients before (pre-CPAP, n = 36) and after CPAP treatment (post-CPAP, n = 36). **(B)** Violin plots illustrating the percentage of CD8^+^ T lymphocytes expressing TIGIT, determined by flow cytometry, in pre- and post-CPAP therapy. Statistical comparisons were performed using the Wilcoxon signed-rank test; p-values are indicated.

Our data showed that the hypoxemia clinical parameters of OSA were associated with TIGIT expression. Thus, we investigated the impact of TIGIT expression after CPAP treatment. TIGIT expression were reduce on CD4^+^ and CD8^+^ T cells after one year of CPAP therapy (**Figure 6**). These results suggest that CPAP treatment is capable to downregulates TIGIT expression.

## Discussion

In this study, we demonstrate that the inhibitory immune checkpoint receptor TIGIT is overexpressed on circulating T cells from patients with severe obstructive sleep apnea (OSA) in the absence of clinically evident cancer. Importantly, TIGIT expression correlated with hypoxemia-related clinical parameters, reinforcing the concept that intermittent hypoxia is a key driver of immune dysregulation in OSA. Mechanistically, our *in vitro* data indicate that intermittent hypoxia induces TIGIT upregulation through HIF-1α–dependent pathways. Functionally, increased TIGIT expression was associated with altered cytokine production and cytotoxic mediator expression, consistent with a phenotype of T-cell dysfunction. Notably, long-term treatment with continuous positive airway pressure (CPAP) significantly reduced TIGIT expression, highlighting the reversibility of this immune alteration. Altogether, these findings identify TIGIT as a hypoxemia-sensitive immune checkpoint in OSA and suggest a potential link between sleep-disordered breathing, immune exhaustion, and impaired immune surveillance.

Hypoxemia in OSA patients induces a dynamic network in which the immune surveillance plays a central role in modulating cancer risk and aggressiveness, mediated by immune checkpoints (8, 31). Immune evasion is a hallmark of tumor progression, and inhibitory checkpoint represent critical mediators of protumoral immune suppression. In this context, our group and others have previously shown that OSA is associated with the upregulation of several immune checkpoints, including PD-1/PD-L1, TIM-3/Galectin-9, PSGL-1, and the CD36/CD73 adenosinergic axis, all of which are linked to cancer progression (11, 12, 14, 15), epithelial–mesenchymal transition (EMT), transforming growth factor beta (TGF-β), and the hypoxemic microenvironment (32–34). Intermittent hypoxia and oxidative stress characteristics of OSA patients may therefore compromise immune surveillance by promoting an immunosuppressive phenotype in circulating immune cells (35–39). The present study extends these observations by identifying TIGIT as an additional immune checkpoint modulated by hypoxemia in OSA, even before the clinical onset of malignancy.

TIGIT has been extensively studied in cancer and chronic inflammatory settings, where it inhibits T-cell activation, proliferation, and cytokine production (40–42). Its ligands, particularly CD155 and CD112, are highly expressed in many tumor types and contribute to immune evasion and tumor progression (43, 44). Consistently, TIGIT-deficient mice show enhanced antitumor immunity and delayed tumor growth in experimental models (45). However, most evidence regarding TIGIT derives from established cancer contexts, including studies in cervical cancer patients and tumor-bearing animals (46–48). In contrast, our study focused on OSA patients without clinical evidence of cancer, representing a pre-tumoral or high-risk stage. In this setting, we did not observe significant differences in membrane-bound or soluble TIGIT ligands versus control subjects. This observation may be explained by several, non-mutually exclusive mechanism: (i) CD155 and CD112 expression may be largely restricted to tumor cells or tumor-conditioned antigen-presenting cells; (ii) full activation of the TIGIT axis may occur predominantly during tumor progression, whereas OSA represents an early permisisive stage characterized by immune checkpoint priming; or (iii) additional, yet unidentified TIGIT ligands may operate under hypoxemic conditions in non-malignant disease. These hypotheses warrant further investigation.

The association between T-cell functionality and T-cell exhaustion has been demonstrated in several pathological contexts, including viral infections and cancer. For example, in chronic hepatitis, blockade of immune checkpoints can reverse T-cell exhaustion, restoring TNF-α and IFN-γ production in CD4^+^ T cells (49). Additionally, in non-Hodgkin lymphoma, the immune checkpoint TIM-3 expressed on T cells affects IFN-γ production through an independent mechanism (50). Together, these findings suggest that T-cell exhaustion, assessed by immune checkpoint expression, contributes to the downregulation of effector T-cell functions such as TNF-α and IFN-γ cytokine secretion. In addition to functional impairment, T-cell exhaustion is characterized by distinct transcriptional changes, including the upregulation of transcription factors such as TOX and the downregulation of others, such as T-bet (51). In agreement with this, our data show that TIGIT expression in T cells is associated with T-cell exhaustion, characterized by reduced TNF-α and IFN-γ levels. In contrast, the cytotoxicity data suggest that increased TIGIT expression could also indicate features of a senescent phenotype (52).

However, most evidence regarding TIGIT derives from established cancer contexts, including studies in cervical cancer patients and tumor-bearing animals (32). Moreover, experimental data support the notion that immune cells dynamically adapt to changes in their microenvironment (53, 54). Thus, reducing hypoxemic stress through CPAP likely alleviates HIF-1α–driven signaling in circulating T cells, leading to normalization of immune checkpoint expression, including TIGIT.

### Limitations

Our study has several limitations. First, OSA diagnosis was based on validated respiratory polygraphy, a standard clinical tool, but not full polysomnography. Second, we were not able to detect TIGIT ligand expression, which may require a tumor-like microenvironment to be upregulated. Third, our OSA cohort did not include patients with clinical evidence of cancer, limiting our ability to directly link tumor progression with TIGIT expression. Finally, the sample size of CPAP-treated patients was smaller than that of the untreated OSA cohort.

## Conclusions

In summary, our study identifies TIGIT as a hypoxemia-regulated immune checkpoint in OSA and provides mechanistic evidence linking intermittent hypoxia, HIF-1α activation, and T-cell dysfunction. The reversibility of TIGIT overexpression with CPAP treatment highlights the clinical relevance of early and effective OSA management to preserve immune competence. These findings contribute to a better understanding of how OSA may predispose patients to cancer and other immune-related comorbidities by reshaping immune checkpoint landscapes before overt disease develops.

## Data Availability

The original contributions presented in the study are included in the article/**Supplementary Material**. Further inquiries can be directed to the corresponding authors.
