# Supplementary material for: Hypoxemia-induced TIGIT expression in obstructive sleep apnea is reversible with continuous positive airway pressure
Source: Front Immunol. 2026 Mar 10;17:1769874. doi: 10.3389/fimmu.2026.1769874 (PMC13008978; doi:10.3389/fimmu.2026.1769874)
Supplement: Supplementary file 1 [file DataSheet1.pdf]

ONLINE DATA SUPPLEMENT

Hypoxemia-induced TIGIT expression in obstructive sleep apnea is reversible with continuous positive airway pressure

Paula Pérez-Moreno[1,2,3], Elena Díaz-García[1,2], Cristina López-Fernández[1,2], Aldara García-Sánchez[1,4], Eva Mañas[4], Laura Pozuelo-Sánchez[4], María Torres-Vargas[1,2], Elisabet Martínez-Cerón[1,2], Raquel Casitas[1,2], Raúl Galera[1,2], María Fernández Velasco[5,6], Luis del Peso[1,7], Francisco García-Rio[1,2,3], Carolina Cubillos-Zapata[1,2]

[1]Biomedical Research Networking Centre on Respiratory Diseases (CIBERES), Madrid, Spain; [2]Respiratory Diseases Group, Respiratory Diseases Department, Hospital La Paz Institute for Health Research – IdiPAZ, Madrid, Spain; [3]Faculty of Medicine, Autonomous University of Madrid, Madrid, Spain; [4]Servicio de Neumología, Hospital Universitario Ramón y Cajal, Madrid, Spain; [5]Clinical and Invasive Cardiology Research Group (ICCI-PAZ), Hospital La Paz Institute for Health Research– IdiPAZ, Madrid, Spain; [6]Cardiovascular Biomedical Research Centre Network (CIBERCV), Madrid, Spain; [7]Regulation of gene expression by hypoxia, Hospital La Paz Institute for Health Research – IdiPAZ, Madrid, Spain;

Supplementary methods

Study subjects

This study cohort comprised individuals recently diagnosed with severe obstructive sleep apnea (OSA), along with matched non-apnoeic control subjects. Patients were consecutively recruited from the Pneumology Departments of La Paz and Ramón y Cajal University Hospitals (Madrid, Spain), based on an apnea–hypopnea index (AHI) greater than 30 events per hour. Diagnosis was established via respiratory polygraphy, which included continuous monitoring of oronasal airflow and pressure, heart rate, thoracoabdominal respiratory movements, and peripheral oxygen saturation (SaO_2).

The indication for CPAP therapy was established in accordance with the International Consensus Document on Sleep (1). In OSA patients for whom CPAP was indicated, pressure titration was performed via telemonitoring to ensure a residual apnea–hypopnea index (AHI) < 5 events/h. The first 36 patients who achieved one year of CPAP treatment (residual AHI 2 ± 1 events/h; adherence 6 ± 1 h/night) were included in a pre–post evaluation model.

Control subjects (CS), matched for sex and age (±2 years), were randomly selected from the municipal registry of the Madrid metropolitan area. Exclusion criteria included current or prior treatment with CPAP, supplemental oxygen, or mechanical ventilation; a history of respiratory diseases (including chronic obstructive pulmonary disease, asthma, or respiratory failure); any infectious disease within the preceding three months; and the use of inhaled or systemic corticosteroids or other anti-inflammatory medications. Participants were classified as current smokers (daily smokers of any amount), former smokers (individuals who had quit smoking at least six months before inclusion), or non-smokers.

Each control subject was matched to a pair of patients with severe OSA based on the sex and smoking status of the first patient and the mean age of the pair. Control participants were not receiving any pharmacological treatment, and OSA was ruled out by respiratory polygraphy. The study protocol was approved by the Ethics Committee of La Paz University Hospital (reference PI-3646).

Flow cytometry

After the 16-hour incubation, cells were collected and stained with fluorochrome-conjugated anti-human antibodies (listed in Supplementary Table S1) for 30 minutes at 4°C in the dark. Cells

were then washed with phosphate-buffered saline (PBS) containing 1% FBS. For intracellular staining, cells were permeabilized using the Transcription Factor Buffer Set (BD Bioscience, Belgium). Appropriate isotype controls were included in all experiments. Data acquisition was performed using a BD FACS-Celesta flow cytometer (Becton Dickinson, Belgium), and analyses were conducted with FlowJo software version X.0.7 (FlowJo LLC, USA). The gating strategy is depicted in Supplementary Figure 1.

Quantification of plasma ligands

Plasma was isolated using Ficoll-Paque Plus density gradient centrifugation, aliquoted immediately, and stored at −80°C until analysis. Plasma levels of CD155 and CD112 were quantified using commercial human ELISA kits (EH79RB and EH331RB, respectively; Invitrogen, Vienna, Austria) in accordance with the manufacturer’s instructions. All samples were assayed in duplicate. Intra- and inter-assay variability metrics are provided in Supplementary Table S2.

Supplementary tables

Table S1. List of fluorochrome-conjugated anti-human antibodies employed in the flow cytometry analysis.

Target	Fluorochrome	Manufacturer	Reference
**CD3**	BUV395	BD-Biosciences (Eysins, Switzerland)	563546
**CD4**	PerCP-Cy™5.5		566923
**CD8**	BV711		563677
**CD14**	BV510		563079
**TIGIT**	BV650		570378
**Perforin**	BV421		563396
**Granzyme B**	PE-CF594		562462
**T-bet**	PE		561268
**TOX**	PE	Miltenyi Biotec (Bergisch Gladbach, Germany)	130-120-785
**CD155**	PE-Vio 615		130-119-000
**CD112**	PE		130-122-770

Table S2. ELISA kits used in the study.

TARGET	MANUFACTURER	REFERENCE	INTRA-ASSAY CV, %	INTER-ASSAY CV, %	LOW-DETECTION LIMIT
**CD155**	Invitrogen (Vienna, Austria)	EH79RB	<10%	<12%	0.8 ng/mL
**CD112**	Invitrogen (Vienna, Austria)	EH331RB	<10%	<12%	0.12 ng/mL

Supplementary Figures

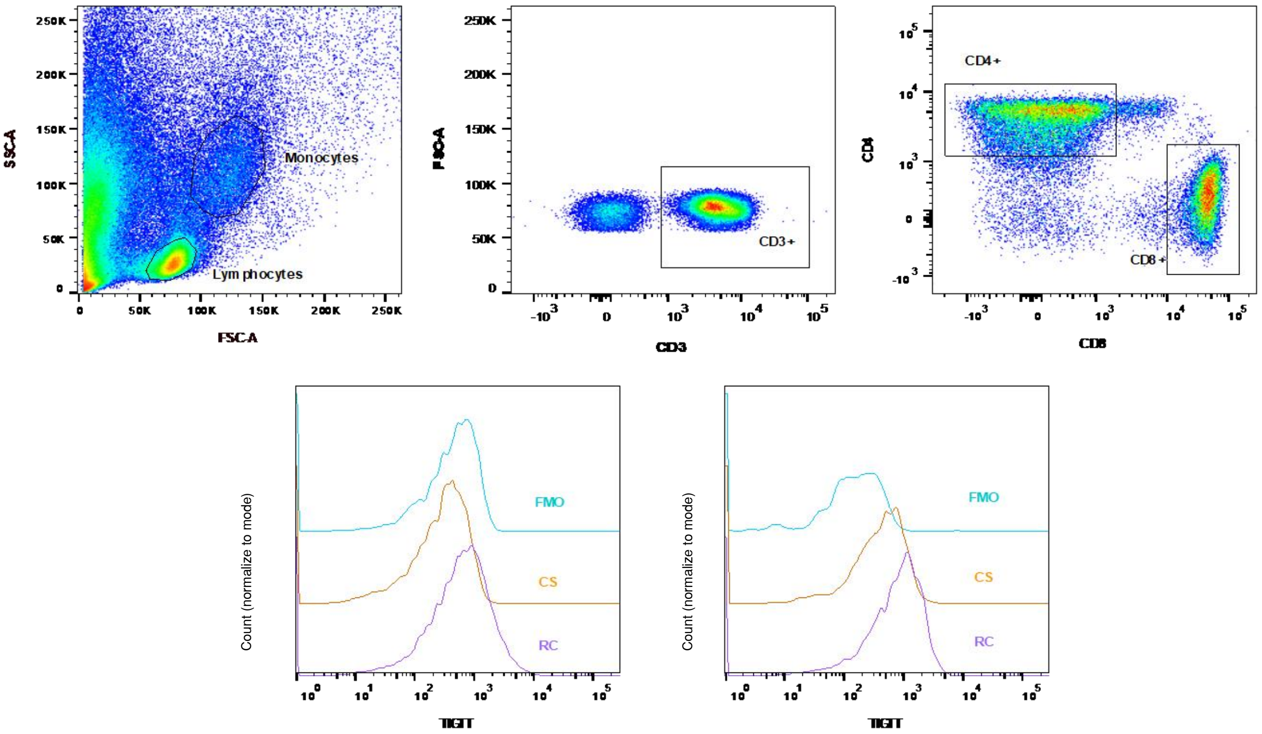

Figure S1: Gating strategy. Gating strategy used to identify CD4⁺ (bottom left) and CD8⁺ (bottom right) lymphocytes expressing TIGIT.

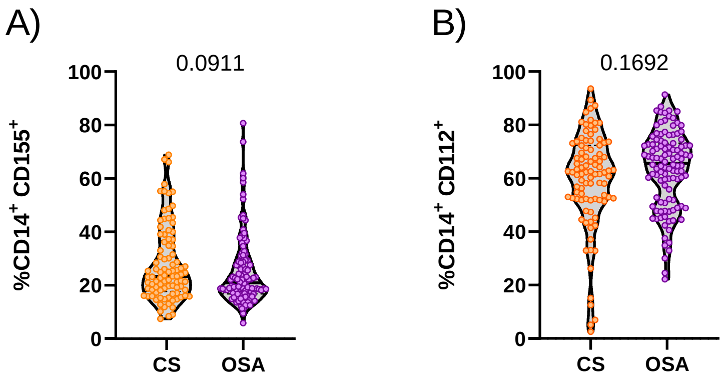

Figure S2: Expression of CD155 and CD112 on Monocyte Membranes. (A) Violin plots depicting the percentage of $CD4^+$ T lymphocytes expressing TIGIT, as determined by flow cytometry, in randomly selected control subjects (n = 92) and patients with obstructive sleep apnoea (OSA) (n = 94). (B) Violin plots showing the percentage of $CD8^+$ T lymphocytes expressing TIGIT, also assessed by flow cytometry, in the same groups. Statistical comparisons were performed using the Mann-Whitney U test; p-values are indicated.

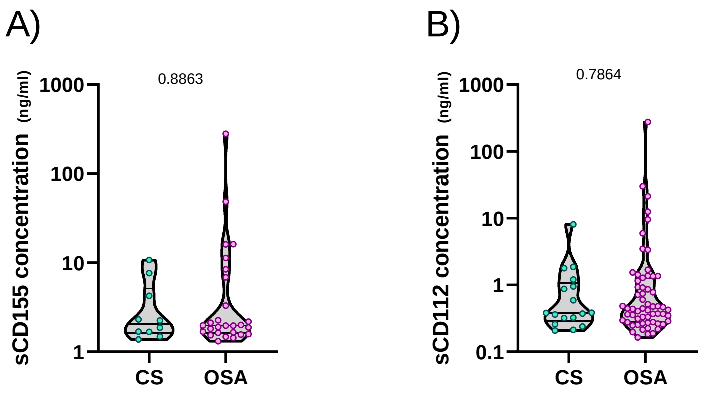

Figure S3: Plasma concentrations of soluble CD155 and soluble CD112. (A) Violin plot illustrating sCD155 levels in control subjects compared to patients with obstructive sleep apnoea. (B) Violin plot illustrating sCD112 levels in the same groups. Statistical comparisons were conducted using the Mann–Whitney U test; *p*-values are indicated.

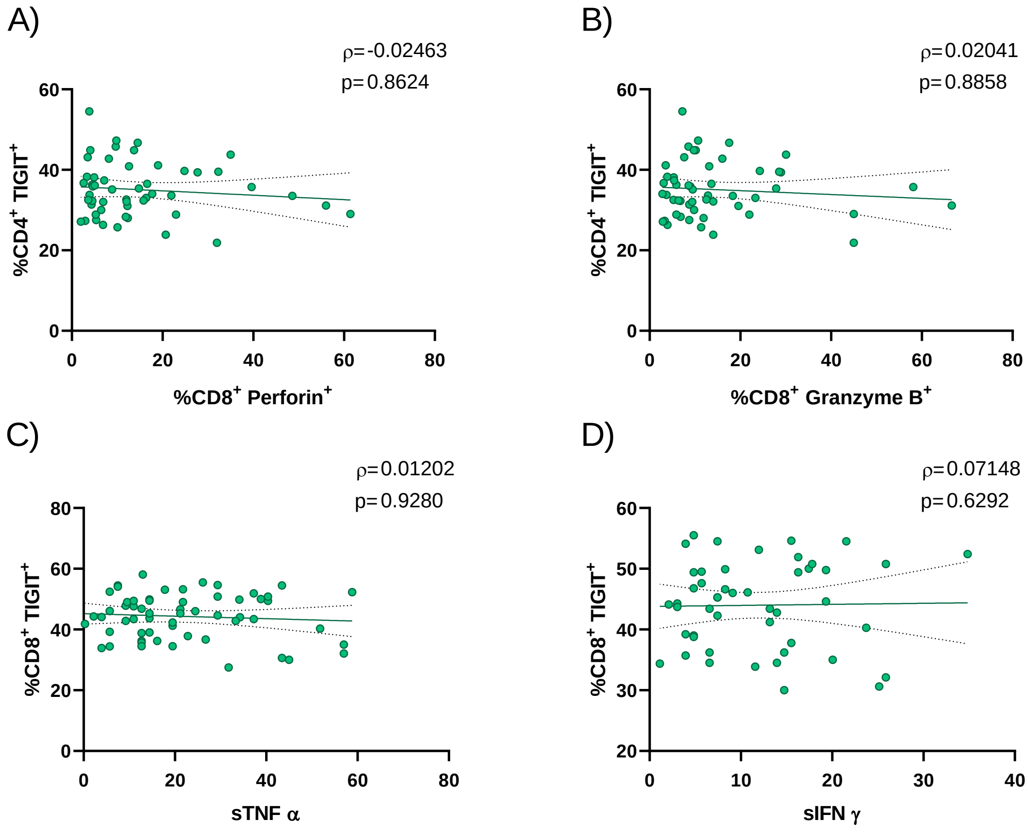

Figure S4: Association between TIGIT expression on CD4⁺ and CD8⁺ T lymphocytes and functional markers. (A-B) Spearman rank correlation analysis between TIGIT expression on CD4⁺ T lymphocytes and the perforin (n=52) (A) and granzyme B (n=52) (B). The solid line indicates the regression trend, while the dashed lines represent the 95% confidence interval. (C-D) Spearman rank correlation between TIGIT expression on CD8⁺ T lymphocytes and soluble TNF-α (n=59) (C) and soluble IFN-γ (n=48) (D). The solid line represents the regression line, and the dotted lines denote the 95% confidence interval.

REFERENCES

1. Mediano O, González N, Montserrat J, Alonso-Álvarezc, L, Almendros I, Alonso-Fernández A, et al. Documento internacional de consenso sobre apnea obstructiva del sueño. Arch Bronconeumol. 2022; 58(1):52-68
